# Nanomedicine and Hyperthermia for the Treatment of Gastrointestinal Cancer: A Systematic Review

**DOI:** 10.3390/pharmaceutics15071958

**Published:** 2023-07-15

**Authors:** Lidia Gago, Francisco Quiñonero, Gloria Perazzoli, Consolación Melguizo, Jose Prados, Raul Ortiz, Laura Cabeza

**Affiliations:** 1 Institute of Biopathology and Regenerative Medicine (IBIMER), Center of Biomedical Research (CIBM), University of Granada, 18100 Granada, Spain; lgago@ugr.es (L.G.); fjquinonero@ugr.es (F.Q.); gperazzoli@ugr.es (G.P.); melguizo@ugr.es (C.M.); roquesa@ugr.es (R.O.); lautea@ugr.es (L.C.); 2 Department of Anatomy and Embryology, Faculty of Medicine, University of Granada, 18071 Granada, Spain; 3 Biosanitary Institute of Granada (ibs.GRANADA), SAS-University of Granada, 18014 Granada, Spain

**Keywords:** gastrointestinal cancer, magnetic nanoparticles, hyperthermia, cytotoxic drugs

## Abstract

The incidence of gastrointestinal cancers has increased in recent years. Current treatments present numerous challenges, including drug resistance, non-specificity, and severe side effects, needing the exploration of new therapeutic strategies. One promising avenue is the use of magnetic nanoparticles, which have gained considerable interest due to their ability to generate heat in tumor regions upon the application of an external alternating magnetic field, a process known as hyperthermia. This review conducted a systematic search of in vitro and in vivo studies published in the last decade that employ hyperthermia therapy mediated by magnetic nanoparticles for treating gastrointestinal cancers. After applying various inclusion and exclusion criteria (studies in the last 10 years where hyperthermia using alternative magnetic field is applied), a total of 40 articles were analyzed. The results revealed that iron oxide is the preferred material for magnetism generation in the nanoparticles, and colorectal cancer is the most studied gastrointestinal cancer. Interestingly, novel therapies employing nanoparticles loaded with chemotherapeutic drugs in combination with magnetic hyperthermia demonstrated an excellent antitumor effect. In conclusion, hyperthermia treatments mediated by magnetic nanoparticles appear to be an effective approach for the treatment of gastrointestinal cancers, offering advantages over traditional therapies.

## 1. Introduction

The treatment of gastrointestinal cancer poses a significant challenge due to its increasing incidence in the population [1]. For example, colorectal cancer (CRC) ranks third in incidence and second in mortality, while stomach cancer (GC) and esophageal cancer (EC) rank fourth and sixth in mortality, respectively [2].

Current treatments encompass resection surgery along with chemotherapy, radiation therapy, and/or targeted therapies [3,4]. A broad range of drugs are utilized in various types of gastrointestinal cancer. These include 5-fluorouracil (5-FU), oxaliplatin (OXA), and irinotecan (IRI) in CRC; 5-fluorouracil combined with leucovorin, docetaxel, and oxaliplatin (FLOT) in GC [5]; and carboplatin and paclitaxel in EC, among others [6]. However, most drugs and their combinations generate side effects that can lead to treatment failure. For example, neurotoxicity is induced by OXA [7], cardiotoxicity by 5-fluorouracil [8], and severe neutropenia and hypersensitivity reactions follow paclitaxel treatment [9]. Additionally, drug resistance mechanisms induced by cytotoxins lead to poor response to chemotherapy and patient relapse [10]. Therefore, there is an essential need for new strategies to improve the prognosis of these diseases.

In this context, nanomedicine has emerged as a promising approach to cancer treatment and diagnosis. Numerous nanoformulations are available, with sizes ranging from 1 to 100 nm. Their inherent characteristics provide a drug delivery system with several advantages, such as reduced side effects of antitumor agents, improved targeting of the affected region, and increased drug levels in the tumor region, among others [10]. Both organic and inorganic nanoparticles (NPs) [11] have been employed to enhance cancer therapy. In fact, lipid-based nanoparticles have been the first clinically approved therapeutic nanoplatform against cancer by the FDA [12]. A classic example of their application is Doxil, a PEGylated liposome loaded with the drug Doxorubicin (DOXO) [13]. More recently, Onivyde, a liposome encapsulated with irinotecan, was approved by the FDA for the treatment of metastatic pancreatic cancer [14].

In this regard, magnetic NPs, a group of inorganic nanoformulations, have been proposed as an innovative strategy due to their physicochemical properties. They consist of a magnetic core and a polymeric coating, with iron oxide NPs being the most widely utilized. Magnetic NPs have superparamagnetic properties, which means that they are magnetized in the presence of an alternating external magnetic field (AMF), but lose magnetization without it, thereby reducing the potential for aggregation in the body and, consequently, the probability of embolization [15,16]. Among the advantages of these NPs is their ability to diffuse to the tumor region due to the application of a magnetic field near the target tissue [17]. Additionally, magnetic cores are used as contrast agents in various imaging techniques, such as magnetic resonance imaging (MRI), or newer techniques such as magnetic particle imaging. This allows for the tracking of these nanoformulations as they circulate within the organism, which is an interesting approach in terms of establishing targeted therapy [18].

Another major advantage derived from the use of magnetic NPs is their ability to generate high temperatures when an AMF is applied, which is known as hyperthermia. This property is considered one of the most intriguing and promising applications in the field of cancer nanomedicine, since it offers the possibility of applying a combined treatment, integrating the antitumor capacity of the drug loaded in the NPs and the hyperthermia generated [19]. Promising results have been obtained in various types of cancer after applying hyperthermia treatment alone or in combination with chemotherapy [20,21]. Recently, Narayanaswamy et al. (2022) utilized NPs with a MnFe_2_O_4_ core and an Fe_3_O_4_ shell against human colon and breast cancer cell lines (MDA-MB-231 and HT-29, respectively), increasing cell death by up to 70% [22]. Furthermore, Piehler et al. (2020) and Rego et al. (2020) demonstrated the applicability of hyperthermia in vivo using DOX-functionalized magnetic NPs and aminosilane-coated iron oxide NPs, respectively [23,24]. Dabaghi et al. (2021) developed 5-FU functionalized chitosan-coated magnetic NPs to deliver hyperthermia specifically against CRC-induced mice, showing a significant reduction in tumor volume and tumor vascularization [25]. In sum, many results support the benefits of hyperthermia therapy in cancer treatment.

In the present systematic review, we analyzed the most recently published studies on the application of hyperthermia based on magnetic NPs in gastrointestinal cancers. The review highlights crucial aspects of the emerging advancements in magnetic nanomaterials and provides a brief overview of the challenges and limitations of this therapeutic strategy.

## 2. Materials and Methods

### 2.1. Study Eligibility

The purpose of the present systematic review was to analyze the most recent and representative information on studies evaluating the therapeutic efficacy of NP-mediated hyperthermia in the treatment of various gastrointestinal cancers. This review was conducted following the criteria set out in the PRISMA guidelines [26]. To this end, only studies from the last 10 years were considered, deeming older ones obsolete. According to the Burton–Kebler index for obsolescence [27], more than half of the publications on this subject were included.

### 2.2. Inclusion Criteria

This systematic review included scientific publications between January 2013 and January 2023, with full text available and written in English. We also included works where hyperthermia treatment was applied through the use of an AMF on the NPs of interest as a therapy against any of the known gastrointestinal cancers.

### 2.3. Exclusion Criteria

Studies were excluded if hyperthermia, defined as an increase in temperature, was applied by any method other than the use of a magnetic field, such as water baths, lasers, ultrasound, etc. Furthermore, reviews, meta-analyses, systematic reviews, book chapters, or editorials were not considered for the review.

### 2.4. Data Sources

For the bibliographic search, the electronic databases Pubmed, SCOPUS, and Web of Science were used. The first established medical subject heading (MeSH) terms included: “Colorectal Neoplasms”, “Gastrointestinal Neoplasms”, “Esophageal Neoplasms”, “Intestinal Neoplasms”, “Stomach Neoplasms”, “Cecal Neoplasms”, “Duodenal Neoplasms”, “Ileal Neoplasms”, “Jejunal Neoplasms”, “Nanoparticles”, “Liposomes”, and “Hyperthermia”. The final search equation was ((“Colorectal Neoplasms” [MeSH Terms] OR “Gastrointestinal Neoplasms” [MeSH Terms] OR “Esophageal Neoplasms” [MeSH Terms] OR “Intestinal Neoplasms” [MeSH Terms] OR “Stomach Neoplasms” [MeSH Terms] OR “Cecal Neoplasms” [MeSH Terms] OR “Duodenal Neoplasms” [MeSH Terms] OR “Ileal Neoplasms” [MeSH Terms] OR “Jejunal Neoplasms” [MeSH Terms]) OR ((“colon” [Title/Abstract] OR “colorectal” [Title/Abstract] OR “colonic” [Title/Abstract] OR “Gastric*” [Title/Abstract] OR “Gastrointestinal” [Title/Abstract] OR “Esophageal*” [Title/Abstract] OR “Intestinal*” [Title/Abstract] OR “Stomach*” [Title/Abstract] OR “Cecal*” [Title/Abstract] OR “Duodenal*” [Title/Abstract] OR “Ileal*” [Title/Abstract] OR “Jejunal*” [Title/Abstract]) AND (“cancer*” [Title/Abstract] OR “tumor*” [Title/Abstract] OR “tumour*” [Title/Abstract] OR “neoplasm*” [Title/Abstract] OR “carcinoma*” [Title/Abstract]))) AND (“nanoparticles” [MeSH Terms] OR “nanoparticle*” [Title/Abstract] OR “nanoconjugate*” [Title/Abstract] OR “liposomes” [MeSH Terms] OR “liposome*” [Title/Abstract]) AND (“hyperthermia” [MeSH Terms] OR “hyperthermia*” [Title/Abstract]). Some minor modifications were made to adjust the search in the rest of the databases.

### 2.5. Study Selection

Two of the authors (L.G. and F.Q.) conducted the literature search. Initially, all articles were analyzed by title and abstract, with those meeting the inclusion criteria being selected. Both authors then reviewed all the selected articles through full-text analysis, considering the established inclusion and exclusion criteria.

### 2.6. Data Extraction

Following the study selection process, the same two authors separately analyzed the selected articles to extract data. The Cohen’s kappa statistical test result exceeded 0.8 (Cohen, 1968), indicating good agreement between the two authors [28]. All discrepancies were resolved by consensus between authors F.Q. and L.G. and, when necessary, two other authors intervened. A specific questionnaire, divided into two evaluation phases, was used to establish the quality of the selected articles; those papers scoring less than 6 points were excluded from the systematic review. Table 1, which presents the data obtained after exhaustive analysis of each article, includes information on the types of nanoformulations used, the antitumor agents transported, the applied magnetic fields, and notable in vitro and in vivo results, in addition to the article references.

## 3. Results and Discussion

### 3.1. Study Description

**Figure 1:**
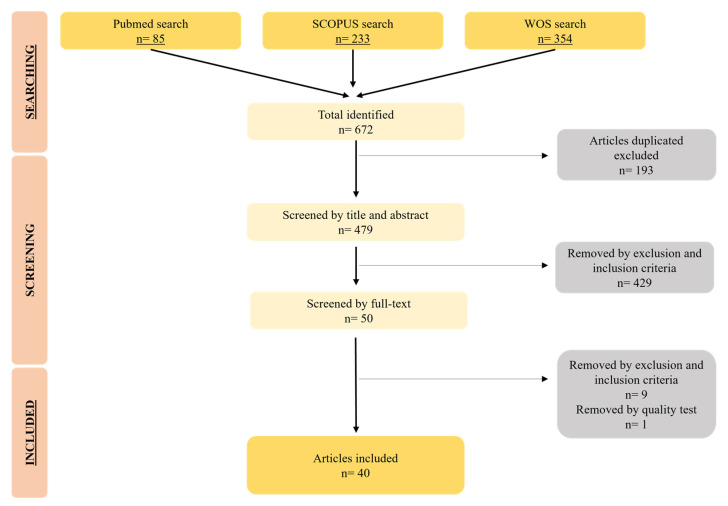
Flow diagram that represents the articles included in the systematic review.

After conducting the bibliographic search in the PubMed, SCOPUS, and Web of Science databases, a total of 672 articles were obtained. Subsequently, 193 duplicate articles were excluded and, once analyzed by title and abstract, another 429 articles were excluded, leaving 50 selected. Likewise, nine of the fifty articles did not meet the inclusion criteria and one of them had low quality values. Therefore, a total of 40 articles were finally included in the present systematic review. All the data concerning the search are represented in the flow diagram in Figure 1.

### 3.2. Characteristics of Magnetic Nanoformulations

**Figure 2:**
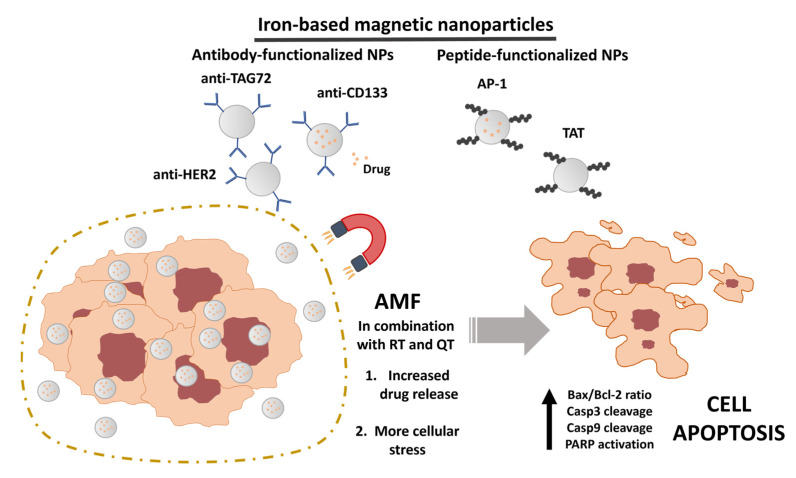
Use of magnetic nanoparticles in in vitro AMF hyperthermia application experiments. Magnetic nanoparticles, typically composed of iron, can be functionalized with antibodies (against HER2, TAG72, or CD133) or specific peptides to actively target a tumor population. AMF facilitates, in formulations that encapsulate drugs, a greater drug release into the cell and the induction of heightened cellular stress. These factors ultimately result in the death of tumor cells, triggering the activation of PARP and the cleavage of several caspases to activate the apoptotic pathway.

Table 1 shows the different nanoformulations used in each article and some of their main characteristics. Of the 40 articles analyzed, 100% of the nanoformulations were based on the magnetic properties of iron oxide nuclei or derivatives (magnetite or maghemite), indicating that iron was the preferred material for creating magnetic NPs. Furthermore, 36 out of 40 articles utilized an NP-based nanocarrier, while the remaining four articles employed more complex systems, including an exosome-based system [29], chitosan nanofibers [30], microrobots [31], and induced pluripotent stem cells [32]. Interestingly, three manuscripts featured antibody-functionalized NPs, including anti-CD133 [33], anti-HER2 [34], and radioactively labeled anti-CC49 [35]. Two articles used AP-1 [36] and TAT [37] peptides for functionalization. Regardless, the objective was to enhance the capabilities of the different nanoformulations (Figure 2). Approximately 47% of manuscripts combined hyperthermia therapy with drug usage. The most widely used chemotherapeutic agents were Doxorubicin (DOXO) and 5-Fluorouracil (5-FU), featured in eight and six articles, respectively. Other drugs included Oxaliplatin (OXA), Irinotecan (Iri), Cisplatin (CDDP), Bortezomib, and Niclosamide. Most of the selected articles analyzed the magnetic characteristics of the nanoformulations. Specifically, 25 articles highlighted the specific absorption rate (SAR) or magnetic saturation point (Ms), both of which are closely related to heat generation capacity after the application of an AMF. The value of the applied magnetic field and the duration of its application vary depending on the hyperthermia system. In fact, 30 of the 40 articles employed a field frequency within the range of 100 to 650 kHz. Conversely, two articles applied frequencies below 100 kHz, five used high frequencies such as Jahangiri et al. (2021) (13.56 MHz) [38], and three did not specify the frequency used. Ha et al. (2020) and Wang et al. (2021) demonstrated that functionalizations such as quantum dots [39] or the inclusion of NPs in gels [40] could impede temperature rise, suggesting that the choice of NPs is very relevant. 

Moreover, and regarding the time of the treatment followed, the application of a 30 min treatment predominated, both in vitro and in vivo (13 of the 40 articles). The rest of the studies applied times ranging from 5 to 60 min. Likewise, in the case of in vivo tests, some authors applied a 30 min treatment continued over time, exposing the experimental animals to cycles of 30 min during the days established in each case [32,35,36]. Transfer to the clinic would involve the application of hyperthermia cycles alone or in combination with other treatments, depending on the approach. Due to the malignancy of the tumors and recurrences, it may be necessary to apply treatment cycles every certain time previously established. This is the case of the clinical trial conducted by Johannsen et al. (2005) for prostate cancer, in which patients were exposed to six weekly 60 min hyperthermia treatments [67]. Finally, concerning cell lines on which the NPs were tested, in vitro or in vivo, 38 articles were conducted on colon lines, notably the CT26 murine colorectal carcinoma line (11 articles) and HT29 human colorectal adenocarcinoma (9 articles). Only three of the forty articles used gastric [32,34] and esophageal [41] cancer lines. Therefore, the scarcity of investigations in some gastrointestinal cancers necessitates new research.

### 3.3. Biocompatibility of Hyperthermia Assays

Hyperthermia treatment safety is a major limitation in its clinical application. Interestingly, iron oxide was employed in the generation of NPs in all analyzed articles (40 articles) due to its biocompatibility [68], thereby avoiding damage to healthy cells. Fernández-Álvarez et al. (2021) used a non-tumor fibroblastic line and human blood samples to ensure no effect on normal tissue, erythrocytes, coagulation, and the complement system [42].

In addition, clinically accepted values of magnetic fields have been established, indicating that the product of the frequency and amplitude values must not exceed 5 × 10^9^, as higher values can potentially harm DNA [69]. In fact, only 12 out of the 40 articles used magnetic fields within the clinically accepted range [32,35,40,43,44,45,46,47,48,49,50,51]. Conversely, 12 articles did not provide the necessary information to calculate this value [29,30,33,36,38,39,41,52,53,54,55,56]. Some authors have sought alternatives to generate magnetic NPs through a combustion system and varying concentrations of citric acid. These NPs induced high temperatures following an 87 kHz magnetic field, suggesting that a high Fe^2+^/Fe^3+^ ratio can enhance the hyperthermic capacity of nanoformulations without the need to increase the field frequency [57]. Ninety-five percent of the selected articles with in vivo tests demonstrated that hyperthermia treatment with NP did not induce damage to healthy tissues. In fact, Shen et al. (2019) generated magnetic solid lipid NPs coated with folic acid (FA) and Dextran and performed biocompatibility assays in CT26 colorectal tumor-bearing mice. Following hyperthermia treatment, they analyzed blood values and potential histological damage, obtaining normal ratios in all cases, thus supporting the apparent safety of these treatments in vivo [56]. Furthermore, Fang et al. (2021) demonstrated that magnetic liposomes functionalized with TAT/CSF1R inhibitor did not cause changes in body weight or histopathological damage following hyperthermia treatment in CT26 tumor-bearing mice [37]. However, new nanoformulation approaches are required that allow for an increase in temperature without increasing the frequency and intensity of the applied AMF. In this regard, it has been described that shortening the distance between NPs can enhance temperature rise [70]. Yang et al. (2020) generated magnetic nanoparticles and assembled and packaged them into a magnetic complex, obtaining higher temperature rises at very low frequencies (1.3–1.8 kHz) compared to single NPs [33]. These results have also been supported by other authors, such as Hu et al. (2023), who developed a controlled intracellular aggregation of NPs in acidic environments, obtaining better overall heating results [71].

### 3.4. In Vitro Assays

Of the 40 articles analyzed, 26 conducted cell viability tests applying hyperthermia treatment with or without chemotherapy (Table 1). All studies displayed better results with AMF than without it. However, significant differences were observed in induced cell death relative to the nanoformulations and the applied hyperthermia protocol.

Castellanos-Rubio et al. (2020) underscored the importance of selecting an optimal iron concentration for generating hyperthermia. They noted that at a concentration of 0.25 mg/mL, no significant cell death was observed in the colorectal cancer cell line HCT116. Conversely, at 0.5 mg/mL, cell survival decreased drastically [58]. Similarly, some NP functionalizations not only enhance heating capabilities but also cytotoxic effects in vitro. For example, Teo et al. (2017) generated SPIONs functionalized with 3-aminopropyltriethoxysilane (APTS) and/or protamine sulfate (PRO) loaded with TNF-α. They demonstrated that PRO increases NP toxicity in tumor cell lines, such as HepG2 and SW480, after AMF application compared to an APTS coating [59].

In certain cases, the molecular characteristics of the tumor cell line enable the selection of the appropriate NP functionalization, as demonstrated by Kagawa et al. (2021), who used anti-HER2 antibodies for treating the NUGC-4 cell line from gastric cancer, not showing any toxicity in healthy human fibroblasts due to their selectivity for internalization in cells with high HER2 expression, characteristic of gastrointestinal tumors such as gastric and esophageal tumors [34,72]. Among our selected articles, four reported complete cell death derived from the treatment [34,52,60,61]. Interestingly, three of these articles applied NP functionalized with carboxydextran. Both Fernandes et al. (2021) and Álvarez-Berríos et al. (2014) employed hyperthermia therapies in combination with chemotherapy, demonstrating the synergy that can result from applying both therapeutic approaches. Specifically, Fernandes et al. (2021) used polymer-coated iron oxide nanocubes loaded with DOXO, applying hyperthermia treatment from 10 to 90 min (3 cycles of 30 min) (182 kHz AMF) on patient-derived tumor stem cells (CSCs) [52]. After 24 h of exposure to treatment, more than 50% cell death was observed, reaching 100% at 7 days with a significant increase in the percentage of apoptosis and necrosis. These authors demonstrated that the heat generated by AMFs enhanced drug release and stimulated internalization in cells, thereby sensitizing them to chemotherapy. Similarly, five additional articles demonstrated significant drug sensitization, corroborating the enhanced release of some chemotherapeutics, such as 5-FU, OXA, and DOXO, following the use of AMF [36,43,46,47,62].

It has been proposed that the improvement following combined hyperthermia-chemotherapy treatment is due solely to the increase in temperature. However, interestingly, three of the selected articles demonstrated that less cell death was induced when water baths were applied (at the same temperature as those induced by AMF) [43,61]. Specifically, Álvarez-Berríos et al. (2013) used cisplatin-loaded iron oxide NPs and increased the temperature using a water bath or 237 kHz AMF. Hyperthermia generated 50% cell death in the Caco-2 CRC cell line compared to the 40% induced by the water bath. They hypothesized that AMF generates additional cellular stress that enhances membrane fluidity and ultimately results in cell death [49]. Therefore, hyperthermia generated by magnetic NPs appears to be a superior option for improving anticancer therapies compared to other systems.

Finally, hyperthermia associated with chemotherapy was not the only therapeutic approach against gastrointestinal cancer. Mirzaghavami et al. (2021) employed a combined treatment of chemotherapy, hyperthermia, and radiotherapy, inducing a greater decrease in the percentage of cell viability (45%) in the colorectal cancer cell line HT29 compared to individual treatments [50]. Additionally, a significant increase in apoptosis and necrosis was observed in the treated cell lines, increasing the Bax/Bcl2 ratio. Hyperthermia therapies induce more pronounced apoptosis than necrosis (Figure 2). Apoptosis was analyzed in nine of the forty selected articles. In fact, Jahangiri et al. (2021) provided an extensive description of this process, noting the overexpression of proapoptotic factor Bax, cleaved caspase-3, cleaved caspase-9, and PARP after treatment on HT29 and HCT116 colorectal cancer cell lines [38]. A similar increase in cleaved caspase-3 was shown in HCT116 by Ahmad et al. (2020) [55]. Moreover, Wydra et al. (2015) observed an increase in the generation of ROS after the application of AMF [63].

### 3.5. In Vivo Assays

A total of 50% (20) of the selected manuscripts carried out in vivo experiments, as shown in Table 1. The most commonly used animals were mice (90%), with CT26 tumor-bearing mice being the cancer model most frequently chosen by the authors. The utilization of magnetic NPs yielded beneficial results in all instances. In fact, Beyk and Tavakoli (2019) utilized nanohybrids of iron oxide and gold NPs, applying a magnet to the tumor region in CT26-tumor bearing mice for 3 h prior to hyperthermia treatment [53]. The results exhibited a higher temperature increase (49 °C) in the tumor area compared to treatment without a magnet (46 °C). This temperature increase led to significant inhibition of tumor size (92%). With regard to the generated temperature, most of the experiments achieved temperatures ranging from 41 to 50 °C [32,60]. However, after removal of the organs and application of MHT, it was observed that in mice with tumors induced from gastric lines, a large increase in temperature (up to 60 °C) was produced by applying ex vivo magnetic hyperthermia for 5 min, while in mice without tumors this high increase occurred in the liver, the organ in which the accumulation of these NPs took place [32]. Garanina et al. (2020) examined the impact of different temperatures on treatment. They noticed effective reduction in tumor growth in CT26 tumor-bearing mice at 42–43 °C. However, in 4T1 breast cancer tumor-bearing mice, which have a more resistant cell line, the tumor cells recurred after 20 days. In this case, effective treatment occurred at 46–48 °C. Furthermore, temperatures of 58–60 °C were tested, but these caused weight and motility losses, although recovery was observed over time [48]. These findings underscore the importance of personalizing treatments based on the tumors to be treated, whenever feasible, and avoiding excessively high temperatures that might lead to adverse effects.

**Figure 3:**
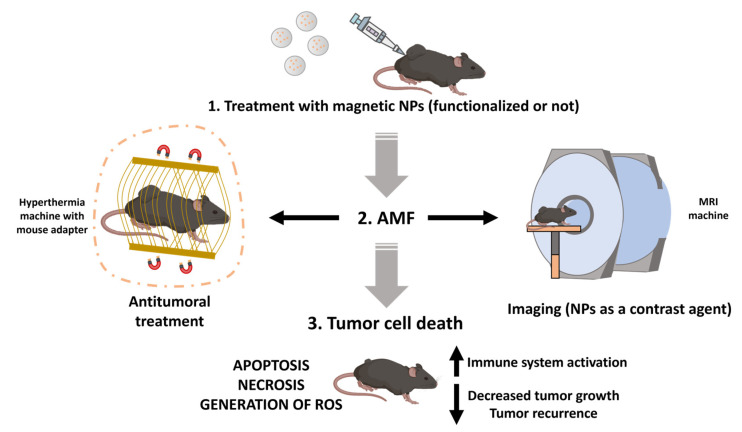
Use of magnetic hypothermia in in vivo experiments. Typically, the assays involve an intravenous administration of the NPs into the mouse such that once they reach the tumor, they are capable of: (1) generating hyperthermia when exposed to an AMF, serving as an antitumor therapy alone or in combination with chemotherapeutic drugs; or (2) acting as contrast agents, creating negative contrast and potentially being used for tumor diagnosis and monitoring. Following the generation of hyperthermia, tumor cell death can occur through several pathways, with apoptosis, necrosis, or extensive oxidative stress being the most notable.

Alternatively, magnetic steering was also reported by Wang et al. (2020) following magnet application (1 h) with positive outcomes [64]. Similarly, Beyk and Tavakoli observed MRI targeting due to these NPs’ ability to act as contrast agents, as was shown in seven other articles (Figure 3) [53]. These results hence signify the possibility of externally enhancing in vivo targeting to the tumor thanks to the magnetic capabilities of NPs. Additionally, Kwon et al. introduced the potential of improving targeting by functionalizing NPs. They applied an FA polymer to the shell of their nanoformulations, achieving improved tumor targeting in HT29 colorectal cancer tumor-bearing mice [29]. However, FA receptors are also found in the small intestine. That is why Shen et al. coated their nanoformulations with dextran, circumventing this compound’s recognition, thus directing more NPs to the colon region where the dextran was degraded by the dextranase produced there [56].

Although most in vivo studies were performed in mice or rats, Liu et al. (2013) conducted an in vivo model of esophageal cancer by injecting VX2 cells into the esophageal mucosa. The authors employed two hyperthermia models: one using a magnetic stem introduced into the esophagus of mice and the other by introducing magnetic NPs into the tumor mass. The results showed that, after the application of a 300 kHz field, both treatments showed a proven anti-tumor efficacy, although it was necessary to control both the temperature and the time of exposure to hyperthermia to avoid causing damage to healthy tissue [41].

All analyzed articles demonstrated positive outcomes in terms of tumor volume reduction when applying hyperthermia and NPs together compared to the application of the AMF or the nanoformulation alone. Nonetheless, it is worth noting that Arriortua et al. (2016) displayed highly varied results, as some tumors in the animal models used (CC-531 colon adenocarcinoma tumor-bearing rats) were almost obliterated while in other cases cell death was minor [65]. Moreover, eight of the twenty-one articles including in vivo analysis examined the apoptotic and necrotic effect induced by hyperthermia at the histological or genetic level. In fact, Beyk and Tavakoli (2019) and Kwon et al. (2021) revealed increases in the expression of some genes indicating apoptosis (cleaved PARP, Bax or cleaved Caspase-3) (Figure 3) [29,53]. These results were confirmed at the histological level in three more articles [35,43,65]. Conversely, Dabaghi et al. (2020) did not demonstrate any modulation in apoptosis gene expression, suggesting a cell death mediated by an increase in ROS [66]. Therefore, the mechanism through which tumor cell death is accomplished could be related to the type of NP and the hyperthermia treatment system. Additionally, three articles assessed the decrease in tumor metastases following treatment. Stankovic’ et al. (2020) did not exhibit dissemination of tumor cells after treatment in histological sections, while Matsumi et al. (2021) and Shen et al. (2019) observed a significant decrease in the number of metastatic nodules and ascites in murine models [35,56,60]. Finally, Fang et al. (2021) transplanted tumor cells from a mouse to other regions post hyperthermia treatment, but no tumor recurrence was noticed, implying activation of immune memory [37]. These results were validated by Jiang et al. (2022) using a CRC model surrounded by bacteria. In this case, immune system activation occurred after hyperthermia, resulting in an increase in cytokines, re-polarization of macrophages, and an increase in antigen presentation [54]. Therefore, hyperthermia treatments also have the ability to activate the immune response, which is typically suppressed in cancer (Figure 3). Likewise, the combination of hyperthermia and immunotherapy is another combined treatment option that may have very promising results. One of the selected articles used a magnetic liposomal system possessing the penetrating TAT peptide by which they administered the CSF1R inhibitor, so that it was possible to repolarize M2 macrophages, thus reducing immunosuppression in the tumor region [37]. It has previously been described that hyperthermia and immunotherapy have synergistic effects, giving rise to the possibility of triggering immunogenic cell death or reversing the immunosuppressive environment of tumors [73]. Thus, after tumor ablation by hyperthermia treatment, antigenic remnants would be released into the environment so they could be used as autologous vaccines against cancer. An example of this is presented in the work carried out by Pan et al. (2020), in which they applied magnetic NPs in combination with the programmed death ligand α-PD-L1 against a breast cancer model. Briefly, the generation of cytotoxic T cells against tumor antigens is achieved and α-PD-L1 prevents tumor immunosuppression, ultimately increasing the number of T cells and the immune response [74]. These results have been confirmed in other articles, demonstrating the potential of this therapeutic approach [75,76].

The benefits observed in in vitro and in vivo trials encourage the transfer of these treatments to the clinic. Currently, the application of magnetic hyperthermia as a possible treatment has been tested in clinical trials against prostate cancer (NCT02033447) and glioblastoma (DRKS00005476). In the first case, patients treated with magnetic nanoparticles received six cycles of therapy for 1 h during phase I of the study and showed tolerance and efficacy as antitumor therapy. Nevertheless, this therapy is still in phase II clinical trials. Regarding the hyperthermia treatment itself, its major limitations lie in the control of the local temperature reached in the tissue, which can negatively affect healthy cells, in addition to the heterogeneous distribution of the temperature in the tumor mass [77]. Additionally, one of the major problems in bringing this therapy to the clinic is the biosafety of the nanoformulations, which must have an exhaustive control of the size and components, making their production totally controlled [69]. Furthermore, another problem is the frequent parenteral administration of nanoparticles, as opposed to the simpler traditional oral administration. This fact generates a more expensive treatment, so that the commercial production of these treatments must be justified by greater efficacy or safety (including side effects) compared to the therapy traditionally used. Finally, many of the results obtained in preclinical studies (in vitro and in vivo models) are not subsequently retained in clinical trials, since certain characteristics, such as specific functionalization against a target, do not act in the same way in these models as in the human body [78].

For all the above reasons, the future of this line of research implies the need to expand the current research in order to solve the drawbacks encountered and finally allow the existence of a novel treatment that improves the quality of life of the affected patients.

## 4. Conclusions

Magnetic NP-driven hyperthermia treatment offers an innovative and promising therapeutic strategy for gastrointestinal cancers. Numerous magnetic NPs, capable of inducing heat and exhibiting varying biological properties, have been developed in recent years. These have been applied to some gastrointestinal cancers, although most assays have been conducted in vitro on CRC. As for the magnetic characteristics of NPs, iron oxide has predominantly been used as the magnetic core, with magnetic fields ranging between 100 and 600 kHz. Nearly half of the tests were conducted using combination therapies with drugs (chemotherapy), with DOXO being notably prominent. The outcomes have been very promising both in vitro and in vivo, reducing metastasis and tumor recurrence in certain cases. However, it has become evident that there is a need to broaden studies to encompass other cancers within the gastrointestinal tract. Further investigation will be necessary to affirm the benefits of hyperthermia application using magnetic NPs in the treatment of gastrointestinal cancer and to overcome barriers to clinical application.

## Figures and Tables

**Table 1:** Summary of the most relevant characteristics of the selected articles.

Nanoformulation	Antitumor Agent	AMF	In Vitro Assay	In Vivo Assay	Tumor Type	Main Results	Reference
MnFe_2_O_4_-Fe_3_O_4_ core–shell NPs	-	384.5 kHz, 27.85 kA/m	Cytotoxicity assay (HT29)	-	CRC	High cytotoxicity effect	[22]
Cs MNPs	5-FU	435 kHz, 15.4 kA/m	-	HT29 tumor-bearing mice	CRC	Decrease in tumor size	[25]
Exosome-FA-MNPs	DOXO	310 kHz	Cytotoxicity assay (HT29)	HT29 tumor-bearing mice	CRC	High cytotoxicity effect and decrease in tumor size	[29]
MNPs loaded Cs nanofibers	-	750–1150 kHz	Cytotoxicity assay (CT26)	-	CRC	High cytotoxicity effect	[30]
SPIONs loaded microrobots	5-FU	430 kHz, 45 kA/m	Cytotoxicity assay (HCT116)	-	CRC	High cytotoxicity effect	[31]
Fluorescent MNP labeled iPS	-	63 kHz, 7 kA/m	-	MGC803 tumor-bearing mice	GC	Decrease in tumor size and good MRI results	[32]
SPIO-APTES anti-CD133 MNPs	IRI	1.3–1.8 kHz	Cytotoxicity (Caco-2, HCT116, DLD1)	HCT116 tumor-bearing mice	CRC	High cytotoxicity assay, decrease in tumor size and good MRI results	[33]
anti-HER2 carboxydextran and amphiphilic polimer SPIONs	-	280 kHz, 31 kA/m	Cytotoxicity assay (NUGC-4)	-	GC	High cytotoxicity effect	[34]
Anti-131I-labeled CC49 SPIONs	-	252 kHz, 15.9 kA/m	-	LS174T tumor-bearing mice	CRC	Decrease in tumor size	[35]
MPVA-AP1 nanovehicles	DOXO	50–100 kHz	Liberation assay	-	CRC	High drug liberation and drug release	[36]
TAT/CSF1R inhibitor functionalized magnetic liposomes	-	288 kHz, 35 kA/m	-	CT26 tumor-bearing mice	CRC	Decrease in tumor size and increased magnetic targeting	[37]
PEG-PBA-PEG coated SPIONs	5-FU	13,560 kHz	Cytotoxicity assay (HT29, HCT116)	-	CRC	High cytotoxicity effect	[38]
Alginate coated MPNPs and QDs	DOXO	4–6.3 kA/m	-	CT26 tumor-bearing mice	CRC	Good MRI results	[39]
Agar encapsulated MNPs	DOXO	400 kHz, 0.45 kA/m	Cytotoxicity assay (HT29)	-	CRC	High cytotoxicity effect	[40]
APTES coated MNPs	-	300 kHz	-	VX2 tumor-bearing rabbits	EC	Decrease in tumor size	[41]
(maghemite/PLGA)/Cs NPs	-	250 kHz, 4 kA/m	Cytotoxicity assay (T84)	Healthy mice	CRC	High cytotoxicity effect and good MRI results	[42]
PLGA SPIONs	DOXO	205 kHz, 2 kA/m	Cytotoxicity assay (CT26)	CT26 tumor-bearing mice	CRC	High cytotoxicity assay, drug release, decrease in tumor size and good MRI results	[43]
Bacteria derived MNPs	-	187 kHz, 23 kA/m	-	HT29 tumor-bearing mice	CRC	In vivo apoptotic and necrotic areas and good MRI results	[44]
Solid-lipid MNPs	-	250 kHz, 4 kA/m	Cytotoxicity assay (HT29)	-	CRC	High cytotoxicity effect	[45]
Bacteria-derived MNPs	5-FU	250 kHz, 4 kA/m	Liberation assay	-	CRC	High drug release	[46]
Bacteria-derived MNPs	OXA	197 kHz, 18 kA/m	Liberation assay	-	CRC	High drug release	[47]
Cobalt ferrite NPs	-	261 kHz, 8–19.8 kA/m	Cytotoxicity assay (CT26)	CT26 tumor-bearing mice	CRC	High cytotoxicity effect and decrease in tumor size	[48]
MNPs	CDDP	237 kHz, 20 kA/m	Cytotoxicity assay (Caco-2)	-	CRC	High cytotoxicity effect	[49]
PEG-PCL-PEG/FA MNPs	5-FU	13,560 kHz, 0.4 kA/m	Cytotoxicity assay (HT29)	-	CRC	High cytotoxicity effect	[50]
MNPs	-	100 kHz, 4 kA/m	MRI assay	-	CRC	Good MRI results	[51]
Iron oxide nanocubes	DOXO	182 kHz	Patient-derived CSCs	Patient-derived CSCs tumor-bearing mice	CRC	High cytotoxicity assay, decrease in tumor size	[52]
Iron oxide NPs/Au NPs core/shell nanohybrid	-	13,560 kHz	Cytotoxicity assay (CT26)	CT26 tumor-bearing mice	CRC	High cytotoxicity effect, decrease in tumor size, increased magnetic targeting and good MRI results	[53]
ZnCoFe_2_O_4_ and ZnMnFe_2_O_4_ NPs	-	1.35 kA/m	Cytotoxicity assay (CT26)	CT26 tumor-bearing mice	CRC	High cytotoxicity effect, decrease in tumor size and better targeting	[54]
Polymers functionalized MNPs	Niclosamide	405 kHz	Cytotoxicity assay (HCT116)	-	CRC	High Cytotoxicity effect	[55]
Magnetic solid lipid NPs coated with FA and Dextran	DOXO	Not specified	Cytotoxicity assay (CT26)	CT26 tumor-bearing mice	CRC	High cytotoxicity effect, decrease in tumor size and metastases	[56]
Acid citric and EDC/NHC functionalized MNPs	-	87 kHz-340 kHz, 79.57 kA/m	Cytotoxicity assay (not specified)	-	CRC	High cytotoxicity effect	[57]
PMAO-PEG MNPs	-	650 kHz, 16.71 kA/m	Cytotoxicity assay (HCT116)	-	CRC	High cytotoxicity effect	[58]
APTS/PRO functionalized SPIONs loaded with TNF-alfa	-	110 kHz, 8.75 kA/m	Cytotoxicity assay (SW480, HepG2)	-	CRC	High cytotoxicity effect	[59]
Carboxydextran coated MNPs	-	390 kHz, 28 kA/m	Cytotoxicity assay (HCT116)	Peritoneal-dissemination mice	CRC	High cytotoxicity effect and metastases decrease	[60]
Carboxydextran coated MNPs	Bortezomib	233 kHz, 29.39 kA/m	Cytotoxicity assay (Caco-2)	-	CRC	High cytotoxicity effect	[61]
Liposome encapsulated citric acid-coated MNPs	DOXO	300 kHz, 59.3 kA/m	Cytotoxicity assay (CT26)	-	CRC	High cytotoxicity effect and drug release	[62]
Monosaccharides coated MNPs	-	292 kHz, 51.0 kA/m	Cytotoxicity assay (CT26)	-	CRC	High cytotoxicity effect	[63]
PLGA SPIONs		930 kHz, 13 kA/m	-	CT26 tumor-bearing mice	CRC	Increased magnetic targeting	[64]
PMAO MNPs	-	606 kHz, 14 kA/m	-	CC-531 tumor-bearing rats	CRC	Heterogeneous cytotoxicity results	[65]
Cs MNPs	5-FU	435 kHz, 15.4 kA/m	-	HT29 tumor-bearing mice	CRC	Sensitizes cells for further therapies and DNA damage	[66]

AP1 (atherosclerotic plaque-specific peptide-1); APTES (3-aminopropyltriethoxysilane); CRC (colorectal cancer); Cs (chitosan); CSCs (Cancer stem cells); CSF1R (Colony stimulating factor 1 receptor); DOXO (doxorubicin); EC (esophageal cancer); EDC (1-ethyl-3-(3-dimethylaminopropyl) carbodiimide); FA (folic acid); 5-FU (5-fluorouracil); GC (gastric cancer); iPS (Induced pluripotent stem cells); IRI (irinotecan); MNPs (magnetic nanoparticles); MPVA (magnetic poly(vinyl alcohol)-based nanovehicles); MRI (magnetic resonance imaging); NHC (N-hydroxysuccinimide); NPs (nanoparticles); OXA (Oxaliplatin); PEG (polyethylene glycol); PLGA (poly(lactic-co-glycolic acid)); PMAO (poly(maleic anhydride-alt-1-octadecene)); SPIONs (Superparamagnetic iron oxide nanoparticles); TAT (Transactivator of transcription peptide).

## Data Availability

Not applicable.
